# Modulation of virulence and metabolic profiles in *Klebsiella pneumoniae* under indole-mediated stress response

**DOI:** 10.3389/fcimb.2025.1546991

**Published:** 2025-02-03

**Authors:** Xueyao Fang, Yanhui Chen, Longhua Hu, Shumin Gu, Junqi Zhu, Yaping Hang, Xingwei Cao, Yanping Xiao, Hong Luo, Chuwen Zhao, Lianhua Xiao, Qiaoshi Zhong

**Affiliations:** ^1^ Jiangxi Province Key Laboratory of Immunology and Inflammation, Jiangxi Provincial Clinical Research Center for Laboratory Medicine, Department of Clinical Laboratory, The Second Affiliated Hospital, Jiangxi Medical College, Nanchang University, Nanchang, Jiangxi, China; ^2^ Department of Marriage and Pregnancy, Ganzhou Zhanggong District Maternal and Child Health Hospital, Ganzhou, Jiangxi, China

**Keywords:** *K. pneumoniae*, indole, fimbriae, metabolism, virulence

## Abstract

Indole, a crucial bacterial signaling molecule, plays a fundamental role in regulating various physiological processes within bacteria, including growth, acid tolerance, biofilm development, motility, and other cellular functions. Its regulatory influence extends beyond indole-producing bacteria, significantly impacting the physiological activities in non-indole-producing species. In this study, we demonstrate that indole enhances the pathogenicity and viability of *Klebsiella pneumoniae* using the *Galleria mellonella* infection model and serum killing assay. Concurrently, indole has varying effects on biofilm formation in *K. pneumoniae*, with some strains showing enhanced biofilm formation ability. To elucidate the underlying molecular mechanisms, transcriptome analysis revealed that indole exposure in *K. pneumoniae* led to the upregulation of genes associated with pili formation and iron acquisition systems, while simultaneously inducing oxidative stress responses. Additionally, our analysis uncovered extensive metabolic remodeling. Specifically, we observed significant upregulation of genes involved in simple carbohydrate utilization pathways, including those responsible for galactose, mannose, and maltose metabolism, as well as enhanced expression of genes associated with pyrimidine biosynthesis. These findings collectively indicate that indole enhances the intestinal colonization and pathogenicity of *K. pneumoniae* primarily by modulation of fimbriae expression and metabolic pathway regulation.

## 1 Introduction

Indole, a molecule produced by various bacteria, plays a crucial role in bacterial cell-to-cell communication and behavioral regulation (Vega et al., 2012). This compound is known to regulate multiple physiological processes within bacteria, including growth, acid tolerance, biofilm development, motility, and other cellular functions (Hirakawa et al., 2009; Lee et al., 2015b; Hashidoko and Kim, 2021). Interestingly, the influence of indole extends beyond indole-producing bacteria, impacting various physiological activities in non-indole-producing species as well. For instance, in *Pseudomonas aeruginosa*, indole and its derivatives suppress motility and virulence factor production while enhancing antimicrobial resistance (Lee et al., 2009). In *Agrobacterium tumefaciens*, a soil-borne bacterium, indole inhibits growth and motility and upregulates genes involved in biofilm formation, which in turn enhances antibiotic tolerance (Lee et al., 2015a). Similarly, in *Klebsiella pneumoniae* (*K. pneumoniae*), indole has been shown to increase antibiotic resistance and promote biofilm formation by modulation of efflux pump activity (Salama et al., 2024). However, despite these observations, the specific molecular mechanisms through which indole regulates virulence factor expression and metabolic pathways in *K. pneumoniae* remain poorly understood.

*K. pneumoniae*, a prevalent clinical pathogen, can cause various severe or potentially lethal infections, including pneumonia, liver abscess, and bloodstream infections, particularly in individuals with compromised immunity or other susceptibility factors. Its pathogenicity is primarily attributed to a variety of virulence factors, including capsular polysaccharides, lipopolysaccharides, pili, and iron acquisition systems (Russo and Marr, 2019). Research has established that intestinal colonization is a critical stage in the progression of *K. pneumoniae*-induced systemic infections (Gorrie et al., 2017). To successfully colonize, *K. pneumoniae* employs sophisticated mechanisms to overcome host immune defenses and microbial competition, involving the coordinated production of multiple bacterial factors, such as colibactin, microcin E492, and salmochelin (Tan et al., 2024). Additionally, key factors such as iron utilization, purine synthesis, fimbriae production, and capsule formation significantly impact its ability to colonize the intestine (Lasaro et al., 2009; Vogel-Scheel et al., 2010; Hsu et al., 2019; Chen et al., 2023; Hecht et al., 2024).

Studies have revealed that indole concentrations in human and mammalian feces range from 0.30 to 6.64 millimolar (Darkoh et al., 2015), levels sufficient to significantly influence the behavior and physiological functions of gut microbiota (Tennoune et al., 2022). The response to indole signaling molecules, however, varies significantly among different bacterial species. For instance, in a *Caenorhabditis elegans* gastrointestinal infection model, indole signaling enhances *Salmonella typhimurium* antibiotic tolerance in the host intestine through the activation of oxidative stress and bacteriophage shock response systems (Vega et al., 2013); however, it exerts inhibitory effects on *Staphylococcus aureus* by suppressing hemolysin production and reducing its invasiveness (Lee et al., 2013). Furthermore, Aman Kumar et al. demonstrated in a mouse model that lower concentrations of indole enhanced the pathogenesis of enteric pathogens enterohemorrhagic *Escherichia coli* and *Citrobacter rodentium*, while higher concentrations suppressed the expression of bacterial virulence genes (Kumar and Sperandio, 2019).

Based on the regulatory functions of indole in intestinal bacteria, we hypothesized that this signaling molecule might modulate the pathogenicity and physiological status of *K. pneumoniae* by influencing its virulence factors and metabolic pathways. This study aimed to investigate the role of indole signaling in *K. pneumoniae* intestinal colonization, with the goal of developing novel therapeutic strategies to control *K. pneumoniae* infections through a better understanding of the molecular mechanisms underlying its effects on bacterial virulence and metabolism.

## 2 Materials and methods

### 2.1 Bacterial strains and materials

The clinical *K. pneumoniae* strain N442 isolated from blood of a patient in the Hepatobiliary Surgery Unit, the Second Affiliated Hospital of Nanchang University, China. *K. pneumoniae* ATCC 700603 was obtained from the China National Health Inspection Center. NTUH - K2044, a highly virulent strain of *K. pneumoniae* isolated from patient with liver abscess, was donated by Professor Fangyou Yu. Indole, dimethylformamide (DMF) were of analytical grade, and purchased from Aladdin (Shanghai, China). Crystal Violet was purchased from Solarbio (Beijing, China). Methanol and glacial acetic acid were purchased from Xilong Chemical (China). *Galleria mellonella (G. mellonella)* larvae were purchased from Huiyude Company (Tianjin, China).

### 2.2 Growth rate measurements

To determine the toxicities of indole, the specific growth rates of the N442 strain, the NTUH-K2044 and the ATCC700603 strain were measured in the different concentrations of indole (0 mM, 0.5 mM, 1.0 mM, 2.0 mM, and 5.0 mM), respectively. Indole was dissolved in dimethylformamide (DMF, ≥99.8%), and 0.5 vol% of DMF was added as a control group at all experiments. All strains were inoculated in LB (Luria–Bertani) broth at 37°C and shaken at 200rpm. Bacterial growth was assessed by monitoring the OD600 values of triplicate samples. Except for the growth rate measurements, a concentration of 0.5 mM indole was selected as the treatment solution in all other experiments.

### 2.3 Biofilm assay

To assess the effect by indole on *K. pneumoniae* biofilm formation, the assay referred to the previous literature with minor adjustments. The overnight bacterial cultures were adjusted to an OD600 of 1 and subsequently diluted 1:100 in 1 mL of LB medium with or without 0.5 mM indole supplementation, and static incubated at 37°C for 48 hours. After the supernatant was discarded, cells were fixed with 99% methanol for 15 min and then aspirated. Place at room temperature until completely dry, and then stained with 1% crystal violet for 15 min. The 24-well plate was washed with running water until colorless, and then eluted with 33% glacial acetic acid, the absorbance was measured at 570 nm (O’Toole and Kolter, 1998). The experiment was repeated for three times, and the average value was calculated for biofilm quantification.

### 2.4 *G. mellonella* infection model

To observe the effect of indole on the virulence of *K. pneumoniae*, the survival of *G. mellonella* larvae were measured, as described earlier with slight modifications (Li et al., 2020). The strains were grown to stationary phase in LB added with or without 0.5mM of indole, then wash the bacterial centrifugation with sterile saline for three times, and diluted to about 1×10^7^ CFU/ml for subsequent injections. The larvae measuring 2 - 2.5 cm in length, weigh 200 - 400 mg, have no black spots on the body surface, show uniform creamy white coloration and display vigorous movement. Each experimental group consists of ten to twelve larvae. A 10-μL bacterial suspension was injected into the body of the larvae via the last left proleg by use of a Hamilton syringe with a 30-gauge needle. The syringes are sequentially flushed with 75% ethanol and sterile saline between strain procedures. After challenge, larvae were placed in petri dish and incubated in dark at 37°C for 3 days. Mortality was observed and recorded every 12 hours. The experiment was repeated for three times in each group.

### 2.5 Serum killing assay

The experiments were performed in accordance with the method described by Juyoun Shin et al (Shin and Ko, 2015). After incubating the strain to be tested overnight, the bacterial concentration was adjusted to 1×10^6^ CFU/ml. Subsequently, 25-μL of the bacterial solution was mixed with 75-μL of serum, thoroughly shaken, and then placed on a constant temperature shaker at 37°C for 3 hours. The LB broth was serially diluted tenfold and plated onto MH plates. The counts of serum-treated bacterial suspensions were determined and compared with the counts of unsaturated bacterial suspensions to calculate the survival rate.

### 2.6 RNA-seq and identification of differentially expressed genes

The strains N442 was cultured in LB supplemented with or without 0.5mM of indole for 16 hours, then transferred to fresh LB and shaken to logarithmic growth phase, and were collected by centrifugation at 3000 × g for 5 min at 4°C. The precipitate was immediately preserved with liquid nitrogen. The bacterial precipitate was sent to Majorbio (Shanghai, China) under dry ice, with three replicates each group. Total RNA extraction, RNA purification, cDNA synthesis, DNA library construction, sequencing and data analyses of the transcriptome were performed by Illumina HiSeq X platform. The database used for alignment was the reference genome of *K. pneumoniae* HS11286 (GCA_000240185.2) obtained from the National Center for Biotechnology Information (NCBI). DESeq2 software was utilized to conduct differential expression analysis on the raw data, and the Benjamini-Hochberg method was used to correct the p-value for multiple tests to obtain the adjusted *p*-value (*P*-adjust). Differentially expressed genes (DEGs) were screened with a *P*-adjust < 0.05 and an absolute value of log2 fold change (log2FC) ≥ 1. Furthermore, RSEM software was employed to quantitatively analyze gene expression, and the quantitative index was TPM (transcripts per million/kilobases). The transcriptome volcano plot and heatmap plot were performed in Hiplot Pro (https://hiplot.com.cn/). The ClusterProfiler R software package was utilized to conduct Gene Ontology (GO) and Kyoto Encyclopedia of Genes and Genomes (KEGG) pathway enrichment analyses of DEGs. The pathway enrichment analysis was assessed using statistical methods based on Fisher’s exact test.

## 3 Results

### 3.1 High concentrations of indole inhibit *K. pneumoniae* growth

**Figure 1:**
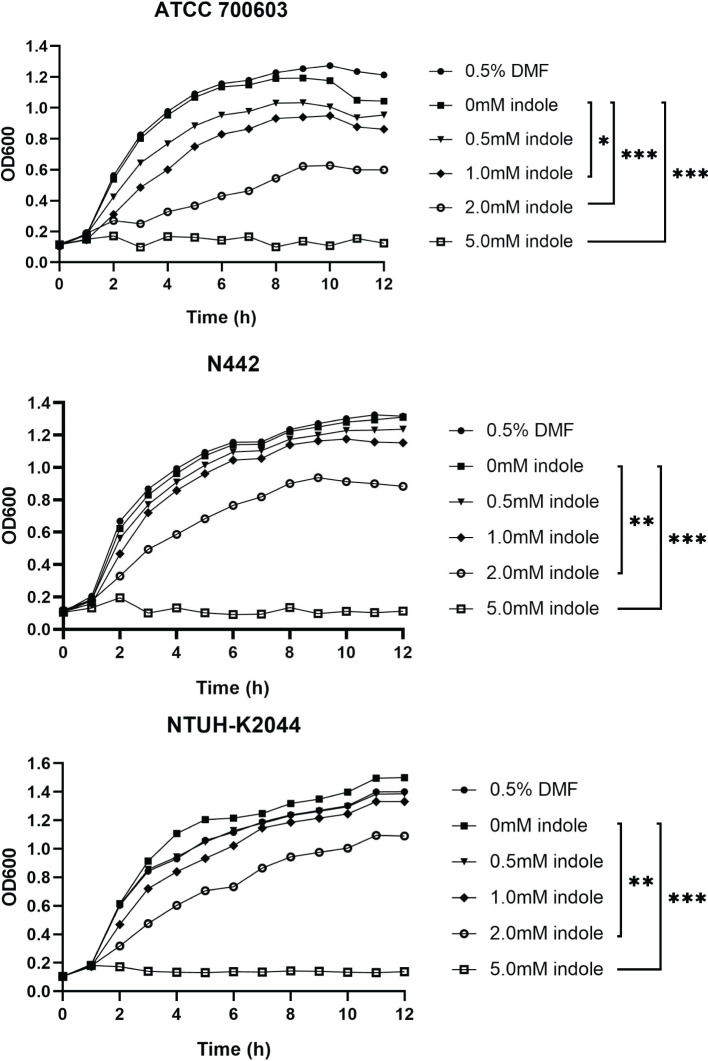
Indole concentration affected the growth of *K. pneumoniae*. Distinct shapes signify the presence of indole solutions with diverse concentrations in the culture medium. P values of each concentration were calculated by comparing growth curves at other concentrations with that at 0mM. Dunnett’s test was utilized for the differential analysis of the growth curves among strains N442, ATCC700603, and NTUH-K2044. Statistical analysis was performed using Two-way ANOVA with Dunnett’s multiple comparison test. “*” means *P*<0.05, “**” means *P*<0.01, “***” means *P*<0.001.

To investigate the effect of indole on *K. pneumoniae* growth, growth curves were generated in LB broth containing various indole concentrations. While all *K. pneumoniae* strains demonstrated growth in indole-containing media, their growth was inhibited at elevated indole concentrations, as illustrated in **Figure 1**. Notably, DMF, used as the indole solvent, showed no effect on *K. pneumoniae* growth. At lower indole concentrations (0.1 mM and 0.5 mM), *K. pneumoniae* exhibited growth patterns comparable to those observed in indole-free conditions, with no statistically significant differences, indicating tolerance within this concentration range. However, when indole concentrations were increased to 1 mM, 2 mM, and 5 mM, bacterial growth was significantly inhibited, with the magnitude of inhibition directly proportional to indole concentration. At 5 mM indole, bacterial growth was severely restricted, approaching a bacteriostatic state.

These findings suggest that *K. pneumoniae* can tolerate indole concentrations between 0 and 0.5 mM, but concentrations exceeding this threshold become increasingly toxic, inhibiting bacterial growth. Based on these results, 0.5 mM indole was chosen as the treatment concentration for subsequent experiments to observe its effects on toxicity, biofilm formation, and transcriptome metabolism at a tolerable indole concentration for *K. pneumoniae*.

### 3.2 Indole’s insignificant effect on *K. pneumoniae* biofilm formation

**Figure 2:**
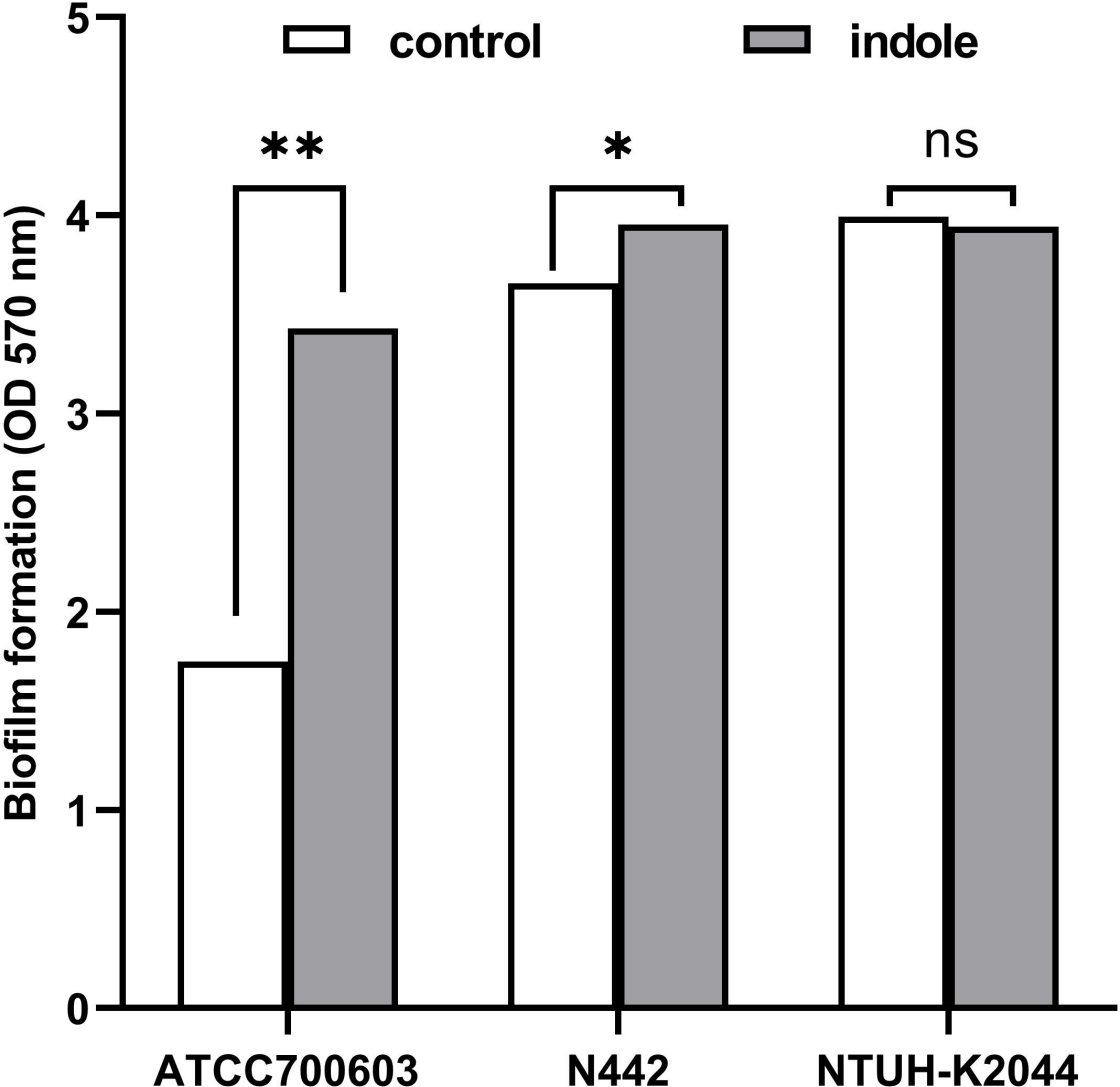
Indole-induced biofilm formation of *K. pneumoniae*. White squares denote bacteria cultured in standard LB broth, while gray squares signify bacteria cultured in broth with 0.5 mM indole. The statistical analysis was conducted using a two-tailed paired t-test. “ns” means *P*>0.05, “*” means *P*<0.05, “**” means *P*<0.01.

Crystal violet staining, a well-established assay for biofilm-forming ability (Muzaki et al., 2024), was employed to assess the impact of indole treatment on biofilm formation in *K. pneumoniae*, as shown in **Figure 2**. The statistical results indicated that indole treatment significantly enhanced the biofilm formation ability of ATCC700603 (*P*=0.0067) and N442 (*P*=0.0105). However, no significant effect was observed on the biofilm formation ability of K2044 (*P*=0.2036). These results imply that indole facilitates biofilm formation within particular strains of *K. pneumoniae*.

### 3.3 Indole could induce enhanced pathogenicity of *K. pneumoniae*

**Figure 3:**
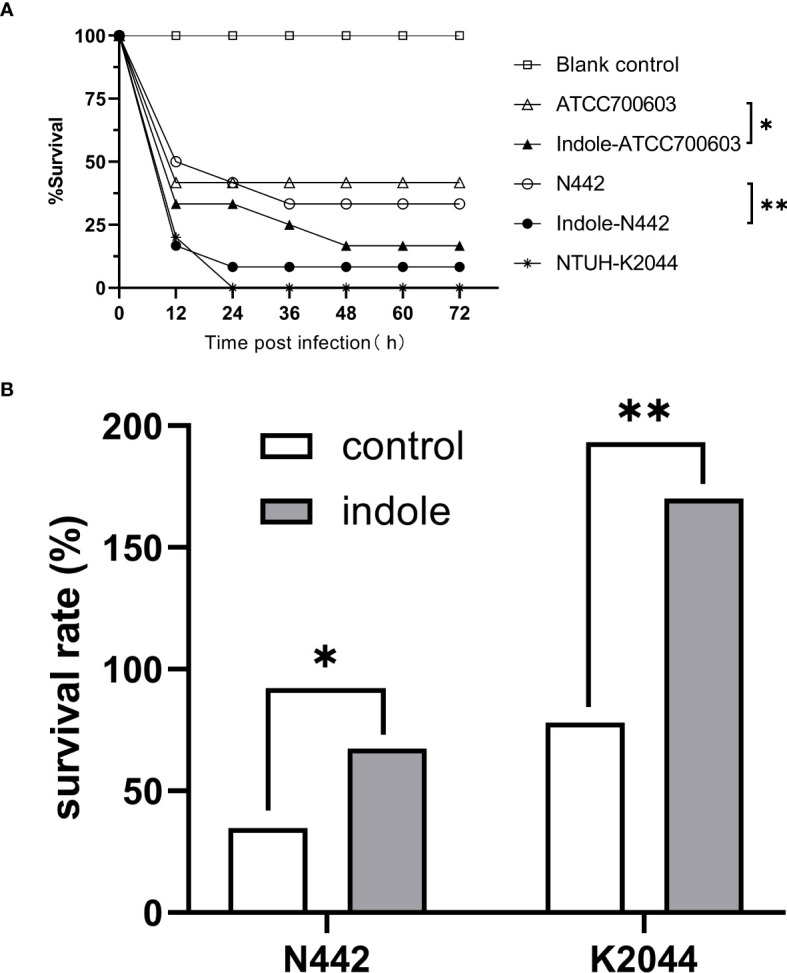
**(A)** Survival rate of *G*. *mellonella* post *K*. *pneumoniae* infection. Different shapes represent the various bacterial suspensions used for injection. Statistical analysis was performed using Two-way ANOVA with Bonferroni correction for multiple comparisons. **(B)** Serum killing assay. The survival rate was calculated through comparing the ratio of colony forming units (CFUs) of bacterial suspensions when serum was added to that of bacterial suspensions in the absence of serum. Statistical analysis was performed using a two-tailed paired t-test. “*” means *P*<0.05, “**” means *P*<0.01.

The *G. mellonella* infection model, which has the capacity to replicate the bacterial infection process within the host organism, was utilized to evaluate the virulence of (D’Apolito et al., 2020). Following exposure to 0.5 mM indole, *K. pneumoniae*-infected larvae showed significantly decreased survival rates, as shown in **Figure 3A**. Specifically, larvae infected with indole-treated N442 (*P*=0.0095) and ATCC700603 (*P*=0.0473) strains exhibited higher mortality rates compared to those infected with untreated *K. pneumoniae*. Using *K. pneumoniae* NTUH-K2044 as a reference strain, the 12-hour mortality rates were significantly elevated in indole-treated strains. The serum killing test is a method of indirectly assessing the resistance of bacteria to the complement system, as shown in **Figure 3B**. In this assay, strains N442 (*P*=0.0032) and K0244 (*P*=0.0293) exhibited significantly higher survival rates compared to the controls lacking indole after 3 hours of incubation in indole-supplemented serum. These results demonstrate that indole treatment significantly enhanced the pathogenicity and viability of *K. pneumoniae* strain N442 in the host.

### 3.4 Indole induces upregulation of fimbriae expression and iron acquisition of *K. pneumoniae* at the transcriptional level

**Figure 4:**
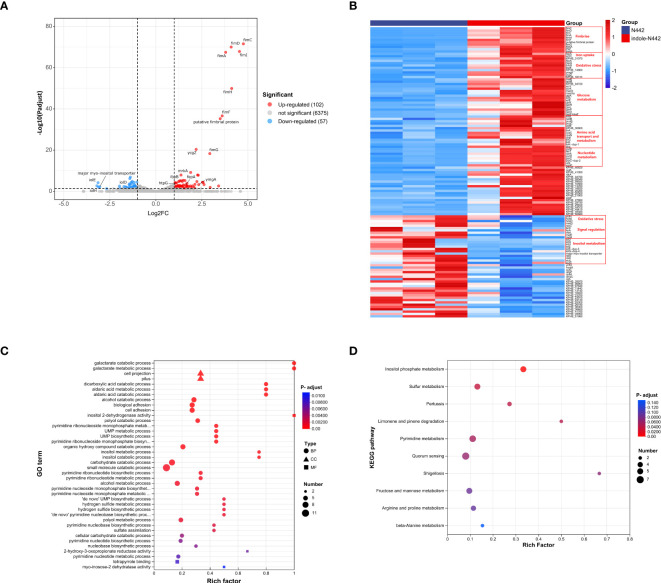
Transcriptome analysis of *K*. *pneumoniae* strain N442. **(A)** Volcano plot depicts up-regulated (red dots) and downregulated (blue dots) DEGs in N442. The thresholds for significant differential expression were set as log2|FC| ≥ 1.0 and *P*-adjust < 0.05. **(B)** Heatmap. Post indole treatment, DEGs of N442 strain were screened for heat map analysis. Upregulated genes in red and downregulated in blue. To ensure the accuracy of the analysis, all small RNA (sRNA) sequences have been excluded from the differential gene list. **(C)** GO enrichment analysis. Different shapes indicate different GO classifications: biological processes (BP) are represented by circles; cellular components (CC) by triangles; and molecular functions (MF) by squares. **(D)** KEGG enrichment analysis. The X-axis represents the Rich factor, which is the ratio of the number of genes in the set of DEGs annotated to the GO term or KEGG pathway to the number of genes in the set of all genes annotated to the GO term or KEGG pathway. The Y-axis represents the top 40 GO terms or top 10 KEGG pathways, sorted by adjusted *p*-values (*P*-adjust), with category names labeled in the figure. The size of the bubbles represents the number of genes enriched in the pathway, while the color of the bubbles indicates the statistical significance of the enrichment, with red representing a small *p*-value (high significance) and blue representing a large *p*-value (low significance).

To investigate changes in gene expression of *K. pneumoniae* in response to indole exposure, RNA-seq experiments were conducted using the clinical strain N442. The transcriptome analysis compared two groups: the experimental group treated with 0.5 mM indole (designated as indole-N442) and the untreated control group (designated as N442). Analysis revealed 159 differentially expressed genes (DEGs) in response to indole treatment, comprising 102 up-regulated and 57 down-regulated genes. Among these DEGs, 34 were sRNAs and 29 encoded hypothetical proteins, while the remaining genes were annotated with defined functions, as illustrated in **Figures 4A, B**.

The proteins encoded by these DEGs significantly influence various vital processes in *K. pneumoniae*, including pili formation, transcriptional regulation, DNA damage repair, and energy metabolism. Notably, genes encoding both type 1 and type 3 fimbriae showed substantial upregulation. The type 1 fimbrial genes exhibited marked increases in expression, including *fimA* (fold change: 13.863, *P*-adjust= 3.45E-68), *fimC* (fold change: 27.041, *P*-adjust= 2.71E-72), *fimD* (fold change: 17.066, *P*-adjust= 9.57E-71), *fimF* (fold change: 12.164, *P*-adjust=2.43E-37), *fimG* (fold change: 7.634, *P*-adjust=4.69E-19), *fimH* (fold change: 17.391, *P*-adjust=1.22E-50), and *fimI* (fold change: 23.319, *P*-adjust=1.38E-68). Similarly, type 3 fimbrial genes showed significant upregulation, including *mrkA* (fold change: 2.579, *P*-adjust=1.21E-08), *mrkB* (fold change: 2.358, *P*-adjust=0.004), and *pilZ* (fold change: 4.949, *P*-adjust=1.51E-08). These fimbriae are known to play crucial roles in host colonization and virulence of *K. pneumoniae* (Alcántar-Curiel et al., 2013).

Four genes associated with iron acquisition demonstrated significant upregulation. *EntC*, which encodes isochorismate synthetase, showed a 3.277-fold increase in expression (*P*-adjust=0.004), while *fepA*, encoding an outer membrane ligand-gated porin, exhibited a substantial 7.634-fold change (*P*-adjust=4.69E-19). These genes work synergistically in the enterobactin synthesis pathway. Additionally, two genes involved in ferrous iron transport were upregulated: *feoA* (fold change: 2.028, *P*-adjust=0.03) and KPHS_31070 (fold change: 2.076, *P*-adjust=0.015). The coordinated upregulation of these iron acquisition genes ensures adequate iron availability for essential bacterial metabolic processes and survival (Kumar et al., 2019).

### 3.5 Indole-triggered oxidative response and transcriptional regulation in *K. pneumoniae*

Indole exposure triggered differential regulation of genes encoding oxidative stress-response proteins and chaperones in *K. pneumoniae*. Notably, two heat shock proteins showed significant upregulation: *htpG*, encoding heat shock protein 90 (fold change: 2.177, *P*-adjust=8.01E-05), and *ibpB*, encoding a small heat shock protein (fold change: 2.454, *P*-adjust=8.89E-06). These proteins play crucial roles in preventing protein degradation under oxidative stress conditions (Grudniak et al., 2015). Additionally, *frmR*, which encodes a formaldehyde-responsive transcriptional regulator, exhibited increased expression (fold change: 2.192, *P*-adjust=0.030). However, several other stress response genes showed downregulation, including *hdeD* (fold change: 0.455, *P*-adjust=0.002), *hokE* (fold change: 0.458, *P*-adjust=0.014), and *msrQ* (fold change: 0.494, *P*-adjust=0.012).

Indole stress also significantly altered the expression of genes involved in quorum sensing and transcriptional regulation in *K. pneumoniae*. The expression levels of several regulatory genes were upregulated, including *ycgZ*, *ymgA*, and two transcriptional regulators: KPHS_14860 (a putative LysR family transcriptional regulator) and KPHS_16110 (a LuxR transcriptional regulator). Conversely, downregulation was observed in *rseC* (encoding a sigma-E factor negative regulator) and two components of the lsr operon: *lsrA* and *lsrC*, which are involved in AI-2-mediated quorum sensing.

### 3.6 Indole-induced metabolic remodeling in *K. pneumoniae*

To elucidate effects of indole on metabolism, we conducted KEGG pathway and GO enrichment analyses. The GO enrichment analysis encompassed 104 differentially expressed genes (DEGs), comprising 66 upregulated (63.5%) and 38 downregulated genes (36.5%) (**Figure 4C**). Among the top 30 enriched GO functional categories, 27 were biological processes (BP), 2 were cellular components (CC), and 1 was molecular function (MF). Within the BP category, 19 significantly upregulated processes were identified, primarily involving carbohydrate and acid metabolism, cell adhesion, pyrimidine biosynthesis, and sulfur-containing compound metabolism. Conversely, 8 significantly downregulated BP categories were identified, predominantly associated with alcohol metabolism, particularly inositol metabolism. In the CC category, upregulation was observed in fimbriae-like proteins and cell projections. The sole enriched MF category showed downregulation of inositol 2-dehydrogenase activity.

KEGG metabolic pathway enrichment analysis was performed on 89 DEGs, consisting of 60 upregulated (67.4%) and 29 downregulated genes (32.6%) (**Figure 4D**). The upregulated DEGs showed significant enrichment in multiple metabolic pathways, including sulfur metabolism, limonene and pinene degradation, pyrimidine metabolism, pertussis, shigellosis, and amino acid metabolism. In contrast, downregulated DEGs were predominantly enriched in inositol phosphate metabolism pathways.

## 4 Discussion

As a signaling molecule, indole is prevalent in the gut and significantly influences the growth and metabolic activity of the gut microbial community (Melander et al., 2014; Lee et al., 2015b). Studies have demonstrated that elevated colonization rates of *K. pneumoniae* within the gut are highly correlated with increased risks of host infection (Martin et al., 2016; Chen et al., 2023; Cai et al., 2024). However, the specific role of indole in regulating the growth, metabolism, and virulence of *K. pneumoniae* remains to be fully elucidated. This study investigated the modulatory effects of indole on the virulence and metabolism of *K. pneumoniae*, as well as its potential to promote intestinal colonization.

### 4.1 Effects of growth and virulence

Growth curve experiments demonstrated that indole at a relatively low concentration (0.5 mM) had no significant effect on *K. pneumoniae* growth, consistent with previous studies (Yaikhan et al., 2019). This finding suggests that at such low concentrations, indole may not directly interfere with bacterial multiplication. However, at concentrations of 1 mM and above, bacterial growth was significantly inhibited, with the inhibitory effect intensifying as indole concentrations increased. These results indicate that high indole concentrations may suppress bacterial growth by disrupting cell membranes or interfering with metabolic pathways (Chimerel et al., 2013; Zarkan et al., 2020). This implies that elevated indole concentrations within the gut might restrict the growth and colonization of *K. pneumoniae*, thereby influencing its ecological niche in this environment.

The *G. mellonella* infection model is an effective tool for evaluating the virulence of *K. pneumoniae*, as it is capable of simulating the bacterial infection process within the host (Li et al., 2020; Asai et al., 2023). Concurrently, the serum killing assay functions as a methodological framework for assessing the resistance of bacteria to the complement system (Shin and Ko, 2015; Liu et al., 2019). Induction with 0.5 mM indole significantly reduced the survival rate of *G. mellonella* and enhanced the serum resistance of *K. pneumoniae*. These findings indicate that indole may play a role in the adaptation of *K. pneumoniae* to host immune pressure, potentially enhancing its survival and pathogenicity.

Consistent with the findings of Yaikhan et al (Yaikhan et al., 2019; Tan et al., 2024), the effect of indole on *K. pneumoniae* biofilm formation varied across strains, with strain K2044 showing no significant enhancement. The variations in the responses of distinct strains to biofilm formation may be closely related to factors (Donlan, 2001; Yin et al., 2019) such as their genetic background, metabolic mechanism, surface structure, stress response, and specific response to indole. It is proposed that, as a highly mucoid strain, K2044 inherently synthesizes large amounts of exopolysaccharides and capsule layers (Peng et al., 2018). These surface structures confer upon the strain a strong biofilm-forming capacity, thereby resulting in minimal effects of indole treatment on its biofilm properties. Future studies should explore the interaction between these bacterial traits and environmental factors to uncover additional details of the biofilm formation mechanism.

### 4.2 Transcriptome regulation

Transcriptome analysis revealed that indole significantly modulates bacterial gene expression, particularly affecting genes related to virulence and fitness. Specifically, indole upregulated genes associated with type 1 and type 3 fimbriae formation, iron acquisition systems, and oxidative stress responses. These molecular changes suggest that indole enhances the pathogenicity of *K. pneumoniae* through multiple mechanisms. On one hand, it strengthens the assembly of bacterial surface structures, such as fimbriae and biofilms, thereby enhancing its adhesive and invasive potential in the environment (Struve et al., 2008; Alcántar-Curiel et al., 2013). On the other hand, it boosts iron acquisition and oxidative stress tolerance, promoting bacterial survival and adaptation in the host gut (Mason et al., 1999; Vega et al., 2013; Denby et al., 2016; Genest et al., 2018; Azaharuddin et al., 2023; Kumar et al., 2019).

In particularly, the *ycgZ-ymgABC* operon-related genes manifested a significant up-regulation in expression, further supports role of indole in promoting *K. pneumoniae* adaptation and biofilm formation under environmental stress. Previous studies have demonstrated a close association between the *ycgZ-ymgABC* operon and the regulation of bacterial biofilms under environmental stress (Tschowri et al., 2009, 2012; Kettles et al., 2019). Additionally, pili, especially type 1 and type 3 fimbriae, directly contribute to biofilm formation. Type 1 fimbriae are crucial for bacteria-host cell adhesion and initial biofilm formation, whereas type 3 fimbriae maintain biofilm stability in a fluid shear environment (Alcántar-Curiel et al., 2013).Indole may enhance the adhesion capacity and biofilm-forming propensity of *K. pneumoniae* via modulating the expression of *ycgZ-ymgABC* operon and fimbria, thereby enhancing its adaptability to the host environment.

### 4.3 Metabolic adaptive remodeling

The transcriptome analysis also revealed substantial indole-induced restructuring of metabolic networks of *K. pneumoniae*. Most notably, genes involved in the utilization of specific carbon sources—including galactose, mannose, and maltose—showed significant upregulation. This metabolic adaptation suggests that indole enhances the ability of bacterium to utilize available host nutrients, thereby supporting growth and survival in the intestinal environment (Chang et al., 2004; Hecht et al., 2024). This conclusion is further supported by previous studies in *E. coli*, where enhanced pyrimidine metabolism was shown to maintain bacterial growth under indole stress (Vogel-Scheel et al., 2010). The parallel findings in these related species suggest a conserved mechanism whereby metabolic remodeling contributes to bacterial adaptation and survival.

### 4.4 Limitations and future directions

Targeting the indole signaling pathway represents a promising strategy for treating *K. pneumoniae* infections, given indole’s crucial role in regulating the bacterium’s virulence and metabolism. By modulating indole levels to influence bacterial growth, virulence, and biofilm formation, its pathogenicity in the intestine can be effectively mitigated, thereby reducing the risk of systemic infection. Studies have shown that indole and its derivatives act as modulators of the intestinal microbiota, promoting intestinal health and mitigate the risk of infection, thus offering novel therapeutic approaches that do not rely on traditional antibiotics (Lee and Lee, 2010; Kim and Park, 2015; Qin et al., 2020; Qayed et al., 2021). Future studies should focus on evaluating the safety and efficacy of indole-based therapies and exploring their clinical potential in treating *K. pneumoniae* infections and other microbiome-related diseases.

However, this study has certain limitations. The molecular mechanisms underlying the regulation of *K. pneumoniae* by indole remain incompletely understood, and no animal models were used to simulate the human intestinal environment. Future studies should further investigate the associated signaling pathways and molecular targets, and incorporate *in vivo* models to gain deeper insights into its pathogenesis and therapeutic potential.

## 5 Conclusions

Overall, indole concentrations have a significant impact on *K. pneumoniae* colonization within the intestinal microbiome. We found that indole enhances the adaptability and virulence of *K. pneumoniae* within the gut milieu by potentiation pili formation, iron uptake, and oxidative stress tolerance. Concurrently, indole refines the bacterial proficiency in host nutrient utilization via metabolic remodeling, thereby facilitating bacterial in the intestinal environment. These findings provide new insights into how microbiota-derived indole modulates pathogenic bacterial colonization in the gut.

## Data Availability

The original contributions presented in the study are publicly available. This data can be found here: Sequence Read Archive (SRA) / PRJNA1215061.
